# The Central Hospitals Board

**Published:** 1896-05-23

**Authors:** B. Burford Rawlings


### THE INSTITUTIONAL WORKSHOP.

### THE CENTRAL HOSPITALS BOARD.

By B. BURFORD RAWLINGS.

The publication of the Hon. Sydney Holland's paper dealing with this subject gives evidence of the stedfastness of his purpose, and secures for his proposals a fuller measure of the attention they deserve. Possibly, the object set before himself by the author of a paper dealing with matter so controversial as this is, would be better served if the meeting he addresses had been previously made acquainted with the workings of his mind—the premises he starts from, the arguments he relies upon, and the conclusions at which he has arrived. This course could not weaken the interest of the seriously inclined, and it would add much to the life and utility of the discussion. Opponents might be encouraged to attack, but friends would be in readiness to defend, and the result would be an interchange of carefully-considered views, which could not fail to add something to knowledge. The disappointing fiasco achieved at the Charing Cross meeting, where Mr. Holland's paper was read, appears to have been due partly to errors in tactics, partly to the not unnatural suspicions aroused by the character of the support the proposal received, and perhaps, most of all, to the sense of unreality and uselessness which clung to the proceedings.

Mr. Holland's paper is such as might be looked for from one whose labours in the hospital field entitle him to confidence. It is the reasoning of a friend, alive to what is good in the work, but pleading for something better; conscious of the value of what is being done by independent and competitive action, but dreaming of a grand confluence of energies and a sustained effort in unison which should substitute for the shortcomings and excesses of a fortuitous concourse of institutions the well-adjusted sufficiency and completeness of a disciplined host.

It is to be regretted that Mr. Holland seems disposed to avow a want of full sympathy—perhaps simulated rather than real—for the personal devotion and enthusiasm of hospital workers; that intense love for, and pardonable pride in, their particular institutions he himself has exhibited in so admirable a degree, and without which the voluntary method as it is now understood would disappear in a decade. The conditions are so complex and susceptibilities so sensitive that the utmost delicacy of touch is needed when handling this subject. The want of co-operation and collective action may be manifest, yet we must at once value and admire the *esprit* which enables the workers to make light of difficulty and discouragement, and has done so much to render our hospitals what they are—the best in the world.

More unfavourable to a prospect of agreement was the presence in some force of the party who, being wholly unsympathetic, are out of place where hospital workers are in conference. No one doubts the sincerity of these gentlemen or can object to their passing resolutions of their own. It is because they are so sincerely iconoclastic that their interference is useless in dealing with questions largely wrapped in sentiment, whose intensity and wholesome power they are unable to appreciate.

Mr. Holland may be right in his belief that there is no deep-rooted objection among hospitals of repute to the constitution of some sort of central body, but if ever we are to have a resolution of value and effect upon this vexed question it must be obtained from a meeting composed of representatives accredited by the recognised authorities of hospitals. Obviously, even they could not coerce their subscribers, though they might influence them; but of what avail can anyone suppose it to be for a meeting casually composed, and whose component parts represent nobody but themselves, to pass drastic resolutions which it has not an atom of power to enforce?

Unfortunate as was the immediate fate of Mr. Holland's efforts, it is not to be assumed that they are necessarily doomed to ultimate failure. It was not his paper which was condemned, but the terms of the resolution that followed it. Possibly, in time, Mr. Holland will be satisfied with the fate of his first resolution, however unceremoniously brought about, seeing that, when it is taken alone, it commits him to an attitude altogether different from that he has assumed in his argument, where with righteous disdain he dismisses the claim of the County Council, and, by inference, other unsympathetic bodies, to interfere with voluntary hospitals.

There was no certainty whatever that if the meeting had agreed to the proposition, "That a central board for the supervision of the London hospitals is needed," it would have gone on, as Mr. Holland proposed, to invoke the friendly offices of a reconstituted Hospital Sunday Fund Council. On the contrary, the probability was that if the first resolution had been passed, the second would have been rejected, and what then? A meeting of so-called representatives of hospitals would have recorded an opinion that the institutions they administer need supervision, and they would have left who will to nominate the supervisors.

The worst enemies of the hospitals could desire no more. That it should have been thought possible for hospital workers to acquiesce is instructive. They might, or might not, be ready to welcome the formation of a central body, but *esprit* must have deteriorated into demoralization before they could be expected to assent to the crude, unqualified, and damnatory proposition that the hospitals needed it for their supervision.

Mr. Holland says that the Central Board must, above all things, "command the purse strings." We would go further than this, and say they must help to fill the purse. The question is pre-eminently one of finance, because, in the case of institutions dependent upon voluntary support, it is essential not only that those who aspire to take part in the management should be required to bear a share of the burden of maintenance, but that the power of control vested in them should be in the exact ratio of the assistance rendered.

If any other basis of interference is admitted the voluntary system is doomed. It is impossible for an outside body to insist upon managing the affairs of other people who have the power to withhold supplies. Ready means of access are provided for all who wish to co-operate, but they must come in at the gate; they cannot be allowed to climb over the wall.

The scheme put forward at the Charity Organisation Society's own meeting, when resolutions were passed in favour of a polyglot council of some 160 members, contemplated an annual contribution of £20,000 to the hospital funds. That proposal showed some, if only a limited, appreciation of the position; but is the society prepared for the logical issue of this admission? The total expenditure of the London hospitals approaches three-quarters of a million sterling, and the proportion of power to be rightly acquired by the contributors would be as twenty to 750, and no more.

Great mental confusion appears to exist in reference to questions which some, with the arrogance of their experience, may regard as elementary. The difficulties of hospital finance are but lightly reckoned by those who are not called upon to surmount them. As with the Lords' Committee, so with reformers in general—and we cannot omit even Mr. Holland—there is no lack of suggestion for additional expenditure, but not a syllable as to how the money is to be obtained.

Given a Central Board acceptably constituted—and Mr. Holland's inclination seems quite in the right direction—great advantages, financial and general, might be expected to follow, and when it had proved capable of rendering assistance the hospitals could not refuse it a share in the control. That is to say, if anybody can be found to go forth valiantly and slay the dragon of poverty which now threatens the life of the voluntary system, doubtless the hospitals would be ready to honour and obey; upon this understanding—that the government to be set up is a constitutional government, and while dealing out stern justice to what is useless and predatory, it shall carefully foster the liberties of every reputable institution.

